# Clinico-pathologic relationships with Ki67 and its change with short-term aromatase inhibitor treatment in primary ER + breast cancer: further results from the POETIC trial (CRUK/07/015)

**DOI:** 10.1186/s13058-023-01626-3

**Published:** 2023-04-12

**Authors:** Judith M. Bliss, Holly Tovey, Abigail Evans, Chris Holcombe, Kieran Horgan, Elizabeth Mallon, Raghavan Vidya, Anthony Skene, Andrew Dodson, Margaret Hills, Simone Detre, Lila Zabaglo, Jane Banerji, Lucy Kilburn, James P. Morden, John F. R. Robertson, Ian Smith, Mitch Dowsett, the POETIC Trialists

**Affiliations:** 1 Clinical Trials and Statistics Unit (ICR-CTSU), The Institute of Cancer Research, London, UK; 2 Poole Hospital, Poole, UK; 3 Royal Liverpool University Hospital, Liverpool, UK; 4 Department of Breast Surgery, St James’s University Hospital, Leeds, UK; 5 Western Infirmary, Glasgow, UK; 6 Royal Wolverhampton NHS Trust, Wolverhampton, UK; 7 Royal Bournemouth Hospital, Bournemouth, UK; 8 UK NEQAS for Immunocytochemistry and In-Situ Hybridisation, London, UK; 9 Ralph Lauren Centre for Breast Cancer Research, Royal Marsden Hospital, and Breast Cancer Now Centre, The Institute of Cancer Research, London, UK; 10 Royal Derby Hospital, University of Nottingham, Derby, UK; 11 Breast Unit, Royal Marsden Hospital, London, UK

**Keywords:** Ki67, Aromatase inhibitor, Primary breast cancer

## Abstract

**Purpose:**

Ki67 assessed at diagnosis (Ki67_baseline_) is an important prognostic factor in primary oestrogen receptor-positive (ER +) breast cancer. Proportional change in Ki67 after 2 weeks (∆Ki67_2week_) is associated with clinical benefit from endocrine therapies and residual Ki67 (Ki67_2week_) with recurrence-free survival. The aim was to define the association between Ki67_baseline_ and after aromatase inhibitor (AI) exposure ∆Ki67_2week_ and Ki67_2week_ with key prognostic and biologic factors utilising data from the POETIC study.

**Patients and methods:**

In POETIC 4480 postmenopausal patients with primary ER and/or PgR + breast cancer were randomised 2:1 to 2 weeks’ presurgical AI (anastrozole or letrozole) or no presurgical treatment (control). Ki67 was measured centrally in core-cut biopsies taken prior to AI and in core-cuts or the excision biopsy at surgery. Relationships between the Ki67 and biologic factors were explored using linear regression.

**Results:**

Established associations of Ki67_baseline_ with biologic factors including PgR status, tumour grade, tumour size, histological subtype, nodal status, and vascular invasion were confirmed in the HER2- subpopulation. In the HER2 + subpopulation only grade and tumour size were significantly associated with Ki67_baseline_. In control group Ki67_2week_ was 18% lower than Ki67_baseline_ (p < 0.001) when Ki67_2week_ was measured in excision biopsies but not when measured in core-cuts. Median suppression by AIs (∆Ki67_2week_) was 79.3% (IQR: −89.9 to −54.6) and 53.7% (IQR: −78.9 to −21.1) for HER2-negative and HER2-positive cases, respectively. Significantly less suppression occurred in PgR- vs PgR + and HER2 + vs HER2- tumours which remained apparent after adjustment for 2-week sample type.

**Conclusions:**

The magnitude of this study allowed characterisation of relationships between Ki67_baseline_, ∆Ki67_2week_ and Ki67_2week_ with high degrees of confidence providing a reference source for other studies. Lower values of Ki67 occur when measured on excision biopsies and could lead to apparent but artefactual decreases in Ki67: this should be considered when either ∆Ki67_2week_ or Ki67_2week_ is used in routine clinical practice to aid treatment decisions or in clinical trials assessing new drug therapies*.*

**Supplementary Information:**

The online version contains supplementary material available at 10.1186/s13058-023-01626-3.

## Background

The nuclear proliferation marker, Ki67, is measured in many malignancies including primary breast cancer[1]. International efforts have shown progress in standardising its measurement such that its value for aiding clinical practise may be realised [2]. Ki67 analysis in primary breast cancer is known to be a prognostic marker for the > 80% of patients whose breast cancers are ER-positive [3] (ER +). Such an example is its licencing as a companion diagnostic for abemaciclib in the US[4]. Yet, where an individual patient’s Ki67 measurement sits within the distribution of the patient population with similar clinical and pathological characteristics is less well described. For example, how unusual is a Ki67 measurement > 20% for a patient with lobular cancer, especially if this is residually high after short-term exposure to an aromatase inhibitor (AI)? Optimising prognostic tools, which incorporate such biomarker results and illustrate the distribution of biomarkers according to classical clinical-pathological factors, is therefore a high priority so that risk-based decisions can be estimated with confidence for the individual patient*.*

Short-term presurgical treatment of patients with primary breast cancer, particularly those with ER + disease, has become popular to gain insights into drug activity but also for identifying groups of patients who may be candidates for response-adapted therapy[5]. Ki67 is the primary endpoint for the large majority of these studies. The limited size of almost all these studies does not permit confident assessment of the relationship with clinico-pathological factors and commonly measured biomarkers or the impact of such on the pharmacologic effectiveness of presurgical therapy on Ki67*.*

In the large majority of primary ER + breast cancer, Ki67 is markedly suppressed by just 2-week endocrine therapy[6]. We and others have shown that the degree of suppression (∆Ki67_2week_) is predictive of response to prolonged endocrine therapy [3, 7]. For example, in the neoadjuvant IMPACT trial, the mean suppression of Ki67 by anastrozole was significantly greater than that by tamoxifen or the combination of anastrozole and tamoxifen at both 2 and 12 weeks[3]. Similarly, in the parallel ATAC adjuvant trial, anastrozole reduced recurrence to a greater extent than tamoxifen or the combination[8]. Given that the mean Ki67 suppression by each of the patient groups in IMPACT was only slightly more at 12 than at 2 weeks, and that 2 weeks is a common duration for the period between breast cancer diagnosis and surgery, the measurement of this biomarker change within what has become known as the presurgical “window of opportunity” has become a primary endpoint in presurgical studies of novel agents. The measurement of Ki67 after such presurgical treatment also has the potential to be used to triage patients away from endocrine treatment alone in the case of suboptimal response[9]. Of particular note regarding prognosis, the absolute level of Ki67 expression at 2 weeks (Ki67_2week_) was shown to be more strongly related to recurrence-free survival than pretreatment levels (Ki67_baseline_)[10]. This seems likely to be due to Ki67_2week_ integrating the intrinsic prognostic value of Ki67_baseline_ and the improvement in prognosis that is reflected by ∆Ki67_2week_. Some investigators advocate the estimation of complete cell cycle arrest (Ki67 < / = 2.7%) for identifying patients with the best prognosis on endocrine therapy[11]*.*

Evidence to inform whether the gain in prognostic insights from measuring Ki67_2week_ is sufficient to merit routine administration of endocrine therapy prior to surgery has been recently reported in the PeriOperative Endocrine Therapy for Individualised Care (POETIC) trial (ISRCTN: 63,882,543, CRUK/07/015)[12]. This trial randomised over 4,400 UK postmenopausal women with hormone sensitive primary breast cancer to receive a non-steroidal AI (letrozole or anastrozole) for 2 weeks prior to and after surgery or no perioperative endocrine treatment (2:1). The study did not show that perioperative endocrine treatment improved long-term outcomes but did show that Ki67_2week_ < 10% was associated with low risk of recurrence. Ki67 analyses from the trial used a scoring method that has formed the basis for international standardisation[13]. We report here the relationship between Ki67_baseline,_ Ki67_2week_ and ∆Ki67_2week_ with key prognostic and biologic factors_._ While we have shown that the large majority of patients show a reduction in Ki67 after 2 weeks’ treatment with an aromatase inhibitor, the degree of change differs markedly between patients. It is known that suppression is greater in tumours with high ER and PgR and in those negative for HER2[14] but the degree to which these relationships are independent of one another and of commonly measured clinico-pathological features could not be established in the modest sized studies to date. The number of patients included in POETIC enabled to address those issues. We also were able to determine if differences in Ki67 levels according to biopsy type were sufficiently substantial to impact on prognostic estimates and to describe extent of Ki67 suppression achieved according to choice of AI, issues for which there was very limited information to date.

## Methods

The primary clinical results and detailed methods for POETIC have already been reported[12]. Details included here are those pertinent to the current report.

### Patients and procedures

POETIC was a phase III, multicentre, randomised trial for postmenopausal women with ER- or PgR-positive invasive breast cancer. Women were randomised (2:1 allocation ratio) to perioperative therapy with a non-steroidal AI (POAI), anastrozole (1 mg/day) or letrozole (2.5 mg/day) (AI choice determined by centre policy) for two weeks before and two weeks after surgery or no perioperative therapy (control). Subsequent therapy was according to local standard of care. Ki67 was evaluated as a biomarker in relation to its effect on predicting disease outcomes and as a secondary endpoint to assess changes between baseline and surgery. Full details of the design and statistical analysis methods of the main study are available in the main clinical paper[12].

Patients provided written consent for the use of core-cut biopsies taken at diagnosis or, if material was not available at diagnosis, for the taking of a core-cut for the purposes of the trial. Investigators were encouraged to take a further core-cut biopsy at the time of surgery but could alternatively provide a representative paraffin-embedded block. Provision of tissue sections was also acceptable at both baseline and surgery. All samples were fixed in formalin prior to paraffin embedding.

### Ki67 methodology

Ki67 was assessed largely according to the method described in Zabaglo et al.[15] that formed the basis for that method validated by the International Ki67 in Breast Cancer Working Group[13]. Ki67 was visualised immunohistochemically using the MIB-1 monoclonal antibody (Dako UK Ltd) at a dilution of 1:50, staining was performed on an automated staining platform (Dako Autostainer, Dako UK Ltd). For scoring, all stained and unstained invasive tumour nuclei were counted in at least 5 high-power fields; the Ki67 staining index was calculated as the total number of stained nuclei counted/total number of all invasive nuclei counted. Only scores from samples in which there were at least 200 invasive cells in total were accepted. QCs consisting of a TMA of at least six cores in duplicate were included in each batch, and batches were only accepted if the scores met specified criteria of acceptance. Paired baseline and surgical samples were stained in the same batch in almost all cases. Scoring was carried out centrally by a team of nine competency-approved technical staff who sought histopathological advice as necessary and practised comparative quality assurance tests throughout the study; 86% of the scoring was conducted by 4 of the staff. Technicians scoring Ki67 were blinded to the treatment allocation. Fewer surgical samples from control patients were analysed because little extra value was expected from multiple samples in the absence of treatment. Initially, all surgical samples were analysed but from early 2013 a subset of one-third of remaining control patients were selected at random for analysis, while all patients in the treatment group were analysed; this led to approximately 7/9 surgical samples from the whole trial being analysed*.*

### Statistical analyses

Medians and interquartile ranges were used to summarise Ki67_baseline_, Ki67_2week_ and ∆Ki67_2week_. ∆Ki67_2week_ was calculated as 100*((Ki67_2week_ + 0.1)—(Ki67_baseline_ + 0.1))/(Ki67_baseline_ + 0.1). The non-parametric sign-test was used to test whether ∆Ki67_2week_ was different from zero in control group patients.

The relationship between each of Ki67_baseline_, Ki67_2week_ and ∆Ki67_2week_ and key prognostic and biologic factors was assessed using linear regression. For Ki67_baseline_ and Ki67_2week_ an outcome of ln(Ki67 + 0.1) was used. For categorical variables, the model coefficient β indicates the mean difference in ln(Ki67 + 0.1) between a designated group and the reference group (indicated by β = 0). For continuous variables β indicates the mean difference in ln(Ki67 + 0.1) per unit increase. For models of ∆Ki67_2week_ an outcome of log-fold change in Ki67 was used, defined as ln((Ki67_2week_ + 0.1)/(ln(Ki67_baseline_ + 0.1)). A positive value of β indicates a smaller drop in Ki67 from baseline to 2 weeks for the designated group compared to the reference group.

Univariable models were fitted containing only the variable of interest. P values given are for a likelihood ratio test comparing this model with a null model containing no variables. Multivariable models were fitted containing all known prognostic variables listed in the same model. P values given are from a likelihood ratio test comparing this model with a model containing all variables except the one of interest. The multivariable models for Ki67_baseline_ and Ki67_2week_ include all factors listed. Multivariable models for ∆Ki67_2week_ additionally include Ki67_baseline_, and were subsequently adjusted for type of AI (letrozole vs anastrozole) and surgical sample type (excision vs core-cut). Models were also repeated only including variables identified as significant in univariable analyses but parameter estimates were not significantly affected so full models are presented for completeness. No adjustment was made to p values for multiplicity but for each multivariable model the adjusted critical value for each term using a Benjamini–Hochberg correction is presented to assist interpretation. Using this procedure, p values are ranked and adjusted critical values are calculated based on the rank. P values are compared to the adjusted critical values; the largest p value which is smaller than its associated critical value and any p values smaller than this are considered significant.

Analyses were based on the snapshot of the clinical data taken on 6th February 2018, consistent with the main clinical results paper. All analyses were performed using STATA 15.

## Results

**Fig. 1:**
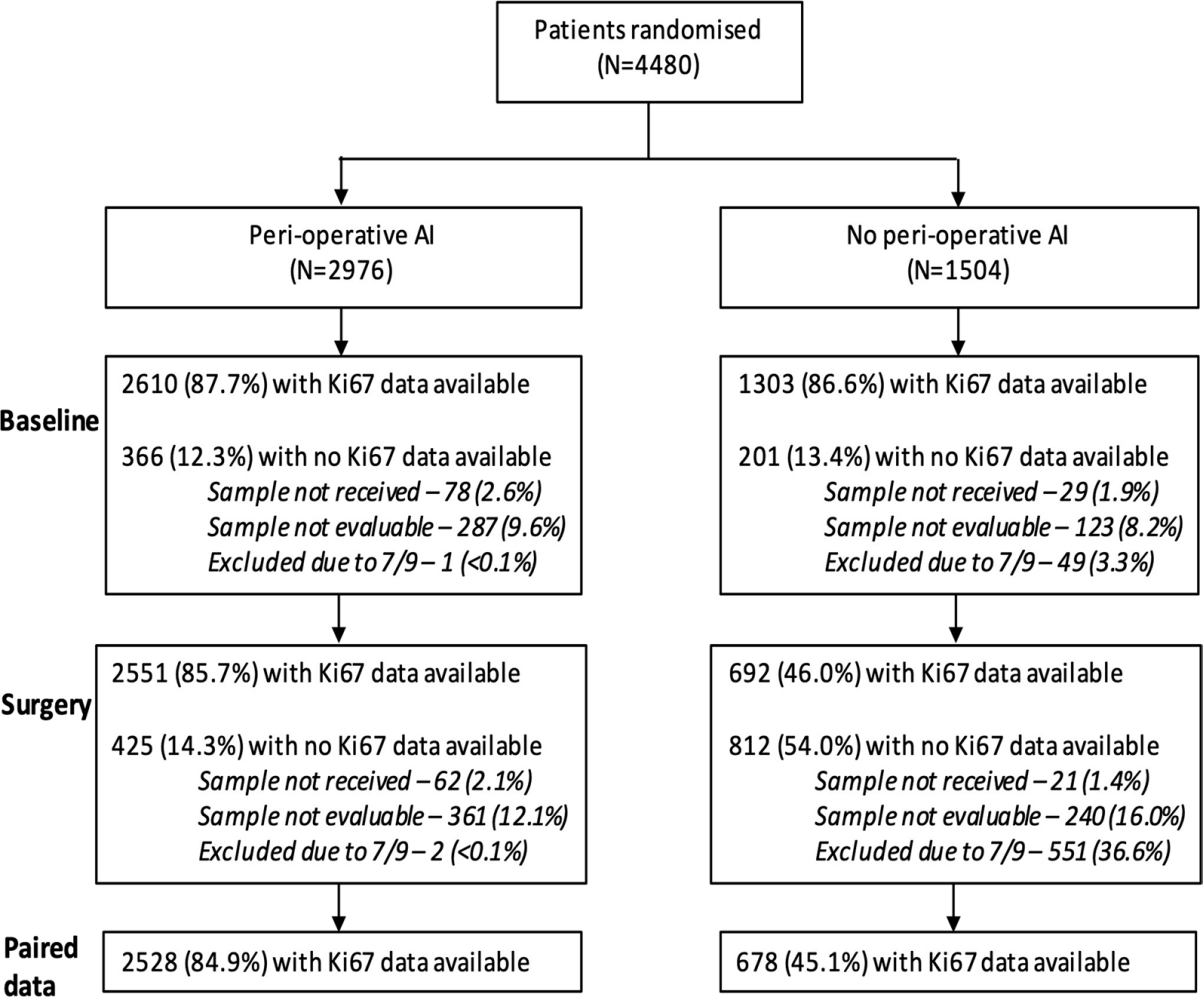
Consort diagram of available samples. Fewer surgical samples from control patients were analysed because little extra value was expected from multiple samples in the absence of treatment. A subset of one-third of control patients were selected at random for analysis, while all patients in the treatment group were analysed; this led to 7/9 samples from the whole trial being analysed

Of the 4480 women (POAI (n = 2976); control (n = 1504)) who entered POETIC, Ki67_baseline_ data were available for 2610 (87.7%) and 1303 (86.6%), respectively; Ki67_2week_ from 2551 (85.7%) and 692 (46.0%); and paired samples to allow calculation of ∆Ki67_2week_ from 2528 (84.9%) and 678 (45.1%), respectively. Figure 1 shows a consort diagram showing reasons for non-availability of data.

### Ki67 assessed at diagnosis (Ki67_baseline_)

**Fig. 2:**
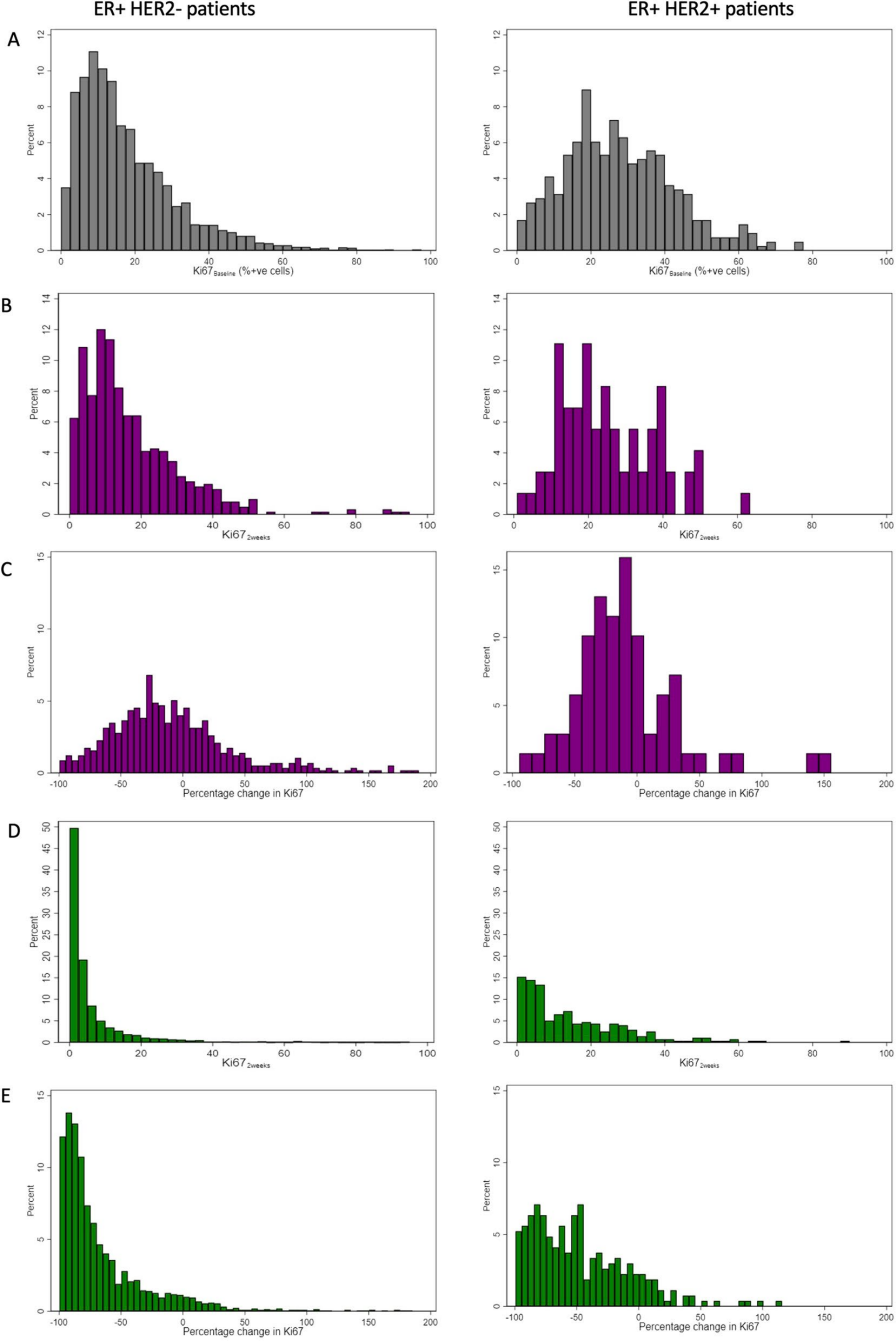
Distribution of Ki67 Distribution of **A** Ki67_Baseline_ for all patients, **B** Ki67_2week_ in patients allocated control, **C** percentage change Ki67 in patients allocated control, **D** Ki67_2week_ in patients allocated AI and **E** Percentage change Ki67 in patients allocated AI. Presented separately for HER2- and HER2 + patients

**Fig. 3:**
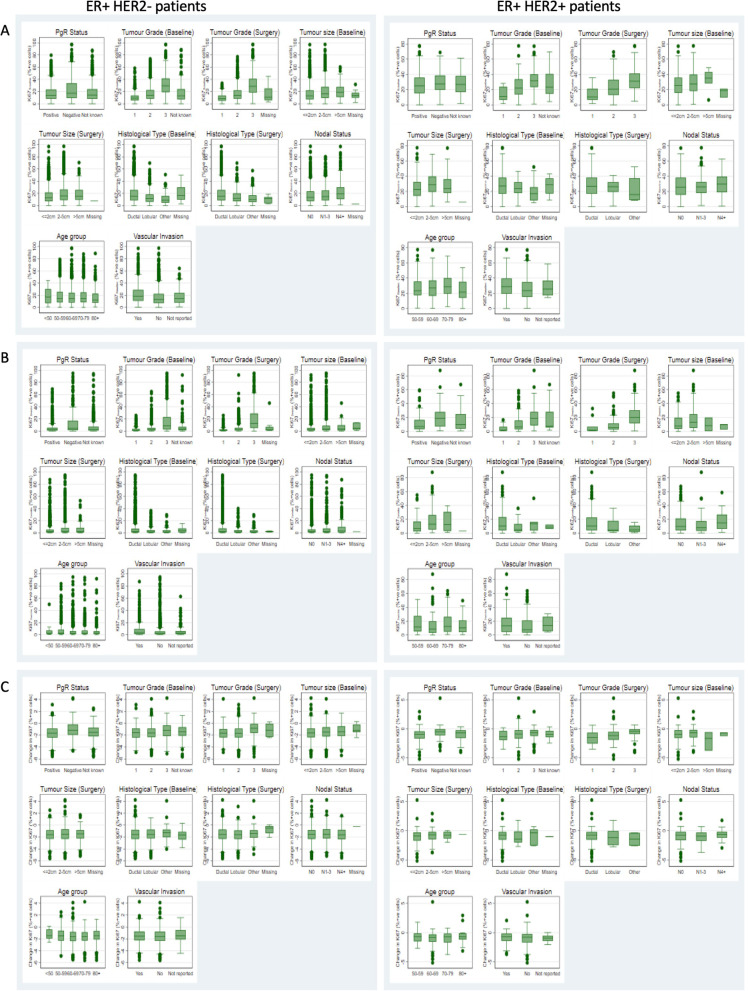
Distribution of Ki67 by clinico-pathological factors Distribution of **A** Ki67_Baseline_ for all patients, **B** Ki67_2weeks_ in patients allocated AI and **C** log-fold change Ki67 in patients allocated AI by clinico-pathological factors. Presented separately for HER2- and HER2 + patients

In this population of 3913 women a highly skewed distribution of Ki67_baseline_ was observed which could be normalised via a logarithmic transformation (Additional file 1: Fig. S1a and b). The median Ki67_baseline_ value was 15.2%; with an IQR of 8.6% to 26.0%; 69.2% of values were above the commonly used threshold of 10%. When considering relationships with common clinico-pathological factors clear evidence was observed of an association with HER2 status (median (IQR) HER2-ve 14.3 (8.2–24.6); HER2 + ve 26.6 (17.0–37.4); Additional file 1: Fig. S1c). Given this finding and the different treatment pathways followed by HER2-ve and HER2 + ve patients all subsequent results are shown for the subpopulations split according to HER2 status, as shown for clinico-pathological factors (Figs. 2a, 3a and Additional file 2: Fig. S2a).

Within the HER2-ve subpopulation (n = 3445) and in univariate analyses a relationship was seen between Ki67_baseline_ and each of the clinico-pathological factors aside from age (Fig. 3a and Table 1). In multivariable analyses a statistically significant association remained for all of these factors (Table 1). This held regardless of whether tumour size was treated as continuous or categorical (additional data not shown).

**Table 1:** Univariable and multivariable linear regression results for Ki67_Baseline_^†^ by HER2 status

	ER + HER2-							ER + HER2 +						
	Univariable			Multivariable				Univariable			Multivariable			
	β	Ci	*p* Value	β	ci	*p* Value	Adjusted critical value	β	ci	*p* Value	β	ci	*p* Value	Adjusted critical value
*PgR Status*														
Positive	0	–	< 0.001	0	–	**0.038**	**0.043**	0	–	0.980	0	–	0.366	0.029
Negative	0.21	0.12–0.31		0.11	0.02–0.20			0.20	0.02–0.38		0.11	− 0.06 to 0.29		
Unknown	0.01	− 0.05 to 0.07		0.00	− 0.06 to 0.06			0.10	− 0.08 to 0.28		0.09	− 0.09 to 0.26		
*Tumour grade (baseline)*														
1	0	–	< 0.001	0	–	** < 0.001**	**0.007**	0	–	< 0.001	0	–	** < 0.001**	**0.007**
2	0.43	0.36–0.51		0.40	0.33–0.48			0.56	0.20–0.92		0.60	0.23–0.97		
3	1.16	1.06–1.25		1.04	0.94–1.14			1.04	0.68–1.40		1.05	0.68–1.42		
Not known	0.40	0.29–0.51		0.35	0.24–0.46			0.69	0.27–1.11		0.74	0.30–1.18		
*Tumour size (baseline)*														
0-2 cm	0	–	< 0.001 (< 0.001)*	0	–	** < 0.001**	**0.021**	0	−	0.167 (0.043)*	0	–	0.561	0.036
2-5 cm	0.24	0.18–0.30		0.14	0.08–0.20			0.14	− 0.01 to 0.29		0.07	− 0.08 to 0.22		
> 5 cm	0.28	0.08–0.49		0.11	− 0.08 to 0.31			0.23	− 0.46 to 0.91		− 0.13	− 0.80 to 0.54		
*Histological type (baseline)*														
Ductal	0	–	< 0.001	0	–	** < 0.001**	**0.014**	0	–	0.286	0	–	0.798	0.043
Lobular	− 0.30	− 0.38 to − 0.22		− 0.24	− 0.31 to − 0.16			− 0.07	− 0.40 to 0.25		0.07	− 0.21 to 0.35		
Other	− 0.50	− 0.65 to − 0.35		− 0.24	− 0.39 to − 0.10			− 0.27	− 1.03 to 0.49		− 0.09	− 0.54 to 0.36		
*Nodal status*														
N0	0	–	< 0.001 (< 0.001)*	0	–	**0.008**	**0.036**	0	–	0.431 (0.211)*	0	–	0.894	0.050
N1-3	0.10	0.03–0.16		0.00	− 0.06 to 0.06			0.06	− 0.11 to 0.24		0.02	− 0.15 to 0.19		
N4 +	0.32	0.23–0.42		0.14	0.05–0.24			0.13	− 0.08 to 0.33		− 0.03	− 0.25 to 0.19		
*Age group*														
< 60	0.02	− 0.06 to 0.10	0.230 (0.059)	− 0.03	− 0.10 to 0.05	0.167	0.050	0.02	− 0.18 to 0.22	0.052 (0.791)*	0.07	− 0.12 to 0.26	0.183	0.021
60–69	0	–		0	–			0	–		0	–		
70–79	0.04	− 0.03 to 0.11		− 0.01	− 0.07 to 0.06			0.15	− 0.03 to 0.34		0.12	− 0.06 to 0.30		
80 +	− 0.07	− 0.16 to 0.03		− 0.10	− 0.19 to − 0.01			− 0.25	− 0.52 to 0.02		− 0.17	− 0.44 to 0.10		
*Vascular invasion*														
Yes	0	–	< 0.001	0	–	** < 0.001**	**0.029**	0	–	0.068	0	–	0.117	0.143
No	− 0.34	− 0.41 to − 0.28		− 0.15	− 0.21 to − 0.08			− 0.17	− 0.32 to − 0.02		− 0.16	− 0.32 to 0.00		
Not reported	− 0.24	− 0.38 to − 0.10		− 0.10	− 0.23 to 0.03			0.06	− 0.39 to 0.51		0.04	− 0.40 to 0.47		

*Test for trend

^†^ Analysed as ln(Ki67 + 0.1)

Adjusted critical values calculated using Benjamini Hochberg method. Significant p values following adjustment are highlighted in bold

Within the smaller HER2 + ve subpopulation (n = 413) in univariate analyses, a relationship was observed between Ki67_baseline_ and grade which remained significant in multivariable analysis. There was also a significant association between Ki67_baseline_ and tumour size treated as ordinal or continuous but this did not remain significant in the multivariable analysis (Fig. 3a and Table 1).

### Ki67_2week_ control group

As expected the logarithmic distribution shown for Ki67_baseline_ was maintained at 2 weeks for patients who were allocated not to receive perioperative AI therapy in both the HER2-ve and HER2 + ve subgroups (Fig. 2b and Additional file 2: Fig. S2b). The median Ki67_2week_ was 13.1% and 23.6% for HER2-ve and HER2 + ve patients, respectively.

#### ∆Ki67_2week_ control group

In the control group for patients with HER2-ve tumours, there was a median fall of 14.6% (IQR: − 40.8–18.3) in Ki67 (∆Ki67_2week_) (Fig. 2C and Additional file 2: Fig. S2C); 100 patients (16.8%) had Ki67_baseline_ ≥ 10% which dropped to < 10% at 2 weeks. In multivariable analyses ∆Ki67_2week_ was associated with Ki67_baseline_ and tumour grade (Additional file 4: Table S1). It was also associated with continuous tumour size but this was not significant in multivariable analyses using the Benjamini Hochberg adjusted critical values and was not significant when categorised.

In HER2 + ve patients, there was a median fall of 12.4% (IQR: − 31.7 to − 7.1) in Ki67; 5 patients (7.1%) had Ki67_baseline_ ≥ 10% which dropped to < 10% at 2 weeks. In univariable analyses, ∆Ki67_2week_4 was associated with Ki67_baseline_ but this was not significant in multivariable analyses after Benjamini Hochberg adjustment to critical values. ∆Ki67_2week_ was not associated with any other clinico-pathological factors in this population (Additional file 4: Table S1).

In order to understand this apparent, potentially artefactual change, analyses of change in Ki67 were explored according to type of sample from which Ki67_2week_ had been calculated. As previously alluded to in the main trial results paper[12] analysis of 679 control group patients with paired samples available (ie Ki67_baseline_ and Ki67_2week_) analyses indicated that where Ki67_2week_ was taken from a core-cut sample the median proportional reduction was − 4·1% (IQR − 27·8 to 34·8), compared to − 17·7% (IQR − 44·2 to 12·7) when a surgical resection sample was used. This significant association between sample type and ∆Ki67_2week_ was observed in the subpopulation of patients with HER2-ve tumours (Additional file 3: Fig. S3a). However, adjusting for sample type in the multivariable model did not materially impact the effect of the clinico-pathological features on ∆Ki67_2week_ (Additional file 4: Table S2). No significant association was observed between sample type and ∆Ki67_2week_ in patients with HER2 + ve tumours.

### Ki67_2week_ POAI group

Following this short exposure to AI treatment the distribution of Ki67_2week_ looked very different to that observed at baseline (Fig. 2d and Additional file 2: Fig. S2d) and the level of Ki67 expression was significantly different. The median was 2.5% (IQR: 1.1–6.5) and 10.3% (IQR: 4.1–21.2) in HER2-ve and HER2 + ve patients respectively with 17.5% of HER2-ve patients and 51.8% of HER2 + ve patients now having Ki67_2week_ above 10%.

In the HER2-ve cohort, the significant univariate relationships seen between grade, tumour size, histologic type (lobular vs ductal), nodal involvement, vascular invasion and Ki67_baseline_ were all observed with Ki67_2week_ (all *p* < 0.001). Effect sizes were similar to those observed with Ki67_baseline_(Fig. 3b and Table 2). PgR negativity was also related to higher Ki67_2week_ and this relationship was stronger than for Ki67_baseline_. Similarly, the contribution of PgR status to the multivariable model was stronger with Ki67_2week_ than with Ki67_baseline_ (Table 2). Tumour size did not remain significant in the multivariable model, while all other relationships were similar for Ki67 assessed at either time-point. This held regardless of whether baseline or surgical grade was used and whether tumour size was considered as categorical or continuous (additional data not shown).

**Table 2:** Univariable and multivariable linear regression results for Ki67_2week_^†^ in patients allocated to AI by HER2 status

	ER + HER2−							ER + HER2 +						
	Univariable			Multivariable				Univariable			Multivariable			
	β	Ci	*p* Value	β	Ci	*p* Value	Adjusted critical value	β	Ci	*p* Value	β	Ci	*p* Value	Adjusted critical value
*PgR Status*														
Positive	0	–	< 0.001	0	–	** < 0.001**	**0.014**	0	–	< 0.001	0	–	**0.004**	**0.014**
Negative	0.70	0.53–0.88		0.49	0.33–0.65			0.75	0.40–1.09		0.50	0.19–0.81		
Unknown	0.18	0.06–0.30		0.11	0.01–0.22			0.27	− 0.06 to 0.60		0.09	− 0.20 to 0.39		
*Tumour grade (baseline)*														
1	0	–	< 0.001					0	–	< 0.001				
2	0.44	0.29–0.59						0.82	0.13–1.52					
3	1.51	1.32–1.70						1.66	0.95–2.36					
Not known	0.63	0.41–0.85						1.36	0.55–2.16					
*Tumour grade (2 week)*														
1	0	–	< 0.001 (0.001)*	0	–	** < 0.001**	**0.007**	0	–	< 0.001 (< 0.001)*	0	–	** < 0.001**	**0.007**
2	0.50	0.37–0.63		0.52	0.38–0.66			0.70	0.03–1.37		0.41	− 0.27 to 1.08		
3	1.96	1.79–2.14		1.85	1.67–2.03			1.88	1.20–2.55		1.44	0.75–2.14		
*Tumour size (baseline)*														
0-2 cm	0	–	< 0.001 (0.001)*					0	–	0.036 (0.066)*				
2-5 cm	0.33	0.22–0.44						0.36	0.07–0.65					
> 5 cm	0.21	− 0.19 to 0.60						− 0.43	− 1.81 to 0.96					
*Tumour size (2 week)*														
0-2 cm	0	–	< 0.001 (0.001)*	0	–	0.224	0.035	0	–	< 0.001 (0.002)*	0	–	0.042	0.021
2-5 cm	0.23	0.12–0.34		0.02	− 0.08 to 0.13			0.63	0.34–0.93		0.34	0.07–0.61		
> 5 cm	0.08	− 0.19 to 0.35		− 0.19	− 0.44 to 0.06			0.49	− 0.19 to 1.18		0.28	− 0.35 to 0.91		
*Histological type (baseline)*														
Ductal	0	–	< 0.001					0	–	0.393				
Lobular	− 0.34	− 0.50 to − 0.19						− 0.41	− 1.02 to 0.19					
Other	− 0.35	− 0.65 to − 0.06						− 0.06	− 0.98 to 0.86					
*Histological type (2 week)*														
Ductal	0	–	< 0.001	0	–	** < 0.001**	**0.021**	0	–	0.198	0	–	0.572	0.043
Lobular	− 0.40	− 0.54 to − 0.25		− 0.30	− 0.44 to − 0.16			− 0.40	− 1.03 to 0.23		− 0.18	− 0.72 to 0.37		
Other	− 0.33	− 0.59 to − 0.07		− 0.04	− 0.28 to 0.21			− 0.80	− 2.00–0.39		− 0.43	− 1.45 to 0.60		
*Nodal status*														
N0	0	–	0.005 (0.002)	0	–	0.782	0.050	0	–	0.058 (0.191)*	0	–	0.420	0.036
N1-3	0.12	− 0.01 to 0.24		0.00	− 0.11 to 0.12			− 0.11	− 0.45 to 0.22		− 0.07	− 0.37 to 0.24		
N4 +	0.28	0.10–0.47		0.06	− 0.12 to 0.24			0.40	0.01–0.79		0.19	− 0.19 to 0.57		
*Age group*														
< 60	0.15	0.00–0.30	0.265 (0.775)*	0.10	− 0.04 to 0.23	0.526	0.043	0.38	− 0.00 to 0.77	0.100 (0.985)*	0.38	0.05–0.71	0.129	0.029
60–69	0	–		0	–			0	–		0	–		
70–79	0.05	− 0.09 to 0.18		0.01	− 0.11 to 0.13			0.37	0.02–0.71		0.15	− 0.15 to 0.45		
80 +	0.05	− 0.13 to 0.23		0.05	− 0.12 to 0.21			0.10	− 0.46 to 0.66		0.19	− 0.29 to 0.68		
*Vascular invasion*														
Yes	0	–	< 0.001	0	–	**0.014**	**0.029**	0	–	0.101	0	–	0.670	0.050
No	−0.40	− 0.52 to − 0.28		− 0.13	− 0.25 to − 0.00			− 0.30	− 0.60 to − 0.01		0.02	− 0.27 to 0.30		
Not reported	− 0.09	− 0.36 to 0.17		0.16	− 0.08 to 0.41			0.12	− 0.80 to 1.05		0.35	− 0.45 to 1.15		

*Test for trend

^†^Analysed as ln(Ki67 + 0.1)

Adjusted critical values calculated using Benjamini Hochberg method. Significant *p* values following adjustment are highlighted in bold

In the HER2 + ve cohort significant univariate associations were observed between Ki67_2week_ and PgR status and grade, both of which remain significant in multivariable analysis (Fig. 3b and Table 2). There was also a significant association between Ki67_2week_ and tumour size, but this only remained significant in multivariable analysis when size was treated as categorical.

### ∆Ki67_2week_ POAI group

The median suppression of Ki67 in relation to baseline was 79.3% (IQR: − 89.9 to − 54.6) and 53.7% (IQR: − 78.9 to − 21.1) for HER2-ve and HER2 + ve cases respectively. The distribution of Ki67 change was logarithmic as shown in Fig. 2e. Only 11.0% of patients did not show a reduction of at least 10% (allowing for variability) after 2 weeks POAI treatment compared to baseline (10.0% & 18.8% for HER2-ve and HER2 + ve respectively).

For both the HER2-ve and HER2 + ve cohorts no significant univariable or multivariable relationship with ∆Ki67_2week_ was observed for tumour size, nodal involvement, histologic subtype or vascular invasion (Fig. 3c and Table 3)_._ However, PgR status and tumour grade were significantly associated with ∆Ki67_2week_ and remained significant in multivariable analysis. Higher Ki67_baseline_ was also significantly associated with a higher proportional change in Ki67 in both cohorts (Table 3). This did not alter following adjustment for sample type in the HER2-ve cohort (Additional file 4: Table S3).

**Table 3:** Univariable and multivariable linear regression results for change in Ki67 (^†^∆Ki67_2week_) in patients allocated to AI by HER2 status

	ER + HER2-							ER + HER2 +						
	Univariable			Multivariable				Univariable			Multivariable			
	β	Ci	*p* Value	β	Ci	*p* Value	Adjusted critical value	β	Ci	*p* Value	β	Ci	*p* Value	Adjusted critical value
Baseline Ki67 (log)	− 0.23	− 0.28 to − 0.17	< 0.001	− 0.41	− 0.47 to − 0.35	** < 0.001**	**0.006**	− 0.35	− 0.50 to − 0.20	< 0.001	− 0.61	− 0.77 to − 0.46	** < 0.001**	**0.006**
*PgR Status*														
Positive	0	–	< 0.001	0	–	** < 0.001**	**0.019**	0	–	0.023	0	–	**0.008**	**0.019**
Negative	0.49	0.34–0.65		0.45	0.30–0.60			0.46	0.13–0.78	–	0.45	0.15–0.75		
Unknown	0.13	0.02–0.23		0.11	0.01–0.22			0.15	− 0.17 to 0.46	–	0.07	− 0.22 to 0.36		
*Tumour grade (baseline)*														
1	0	–	< 0.001					0	–	0.378				
2	− 0.01	− 0.15 to 0.12						0.23	− 0.46 to 0.93					
3	0.30	0.13–0.48						0.45	− 0.26 to 1.15					
Not known	0.19	− 0.01 to 0.39						0.38	− 0.43 to 1.19					
*Tumour grade (2 week)*														
1	0	–	< 0.001 (< 0.001)*	0	–	** < 0.001**	**0.013**	0	–	< 0.001 (< 0.001)*	0	–	** < 0.001**	**0.013**
2	0.01	− 0.12 to 0.13		0.23	0.10–0.37			0.31	− 0.39 to 1.01		0.32	− 0.34 to 0.97		
3	0.68	0.51–0.85		1.16	0.97–1.34			0.84	0.13–1.54		1.14	0.45–1.82		
*Tumour size (baseline)*														
0-2 cm	0	–	0.221 (0.015)*					0	–	0.094 (0.337)*				
2-5 cm	0.09	− 0.01 to 0.19						0.20	− 0.07 to 0.47					
> 5 cm	0.05	− 0.30 to 0.40						− 0.95	− 2.24 to 0.34					
*Tumour size (2 week)*														
0-2 cm	0	–	0.952 (0.601)*	0	–	0.525	0.044	0	–	0.215 (0.249)*	0	–	0.201	0.031
2-5 cm	0.00	− 0.10 to 0.10		− 0.03	− 0.13 to 0.07			0.25	− 0.03 to 0.53		0.23	− 0.03 to 0.50		
> 5 cm	− 0.04	− 0.27 to 0.20		− 0.13	− 0.36 to 0.10			0.19	− 0.46 to 0.84		0.19	− 0.42 to 0.80		
*Histological type (baseline)*														
Ductal	0	–	0.452					0	–	0.639				
Lobular	− 0.02	− 0.16 to 0.11						− 0.22	− 0.78 to 0.34					
Other	0.16	− 0.10 to 0.42						0.22	− 0.64 to 1.07					
*Histological type (2 week)*														
Ductal	0	–	0.252	0	–	0.091	0.031	0	–	0.499	0	–	0.624	0.044
Lobular	− 0.10	− 0.23 to 0.03		− 0.14	− 0.27 to − 0.01			− 0.21	− 0.80 to 0.38		− 0.16	− 0.69 to 0.37		
Other	0.08	− 0.16 to 0.31		0.06	− 0.17 to 0.29			− 0.54	− 1.66 to 0.57		− 0.37	− 1.36 to 0.62		
*Nodal status*														
N0	0	–	0.692 (0.773)*	0	–	0.880	0.050	0	–	0.111 (0.400)*	0	–	0.252	0.038
N1-3	0.03	− 0.08 to 0.14		0.01	− 0.10 to 0.12			− 0.16	− 0.47 to 0.16		− 0.09	− 0.38 to 0.21		
N4 +	− 0.05	− 0.21 to 0.11		− 0.03	− 0.20 to 0.13			0.28	− 0.09 to 0.64		0.23	− 0.14 to 0.59		
*Age group*														
< 60	0.12	− 0.01 to 0.25	0.166 (0.992)*	0.10	− 0.02 to 0.23	0.292	0.038	0.24	− 0.13 to 0.60	0.285 (0.929)*	0.34	0.02–0.66	0.130	0.025
60–69	0	–		0	–			0	–		0	–		
70–79	0.00	− 0.12 to 0.12		0.00	− 0.11 to 0.12			0.14	− 0.18 to 0.46		0.08	− 0.21 to 0.38		
80 +	0.12	− 0.05 to 0.28		0.09	− 0.07 to 0.24			0.45	− 0.08 to 0.97		0.32	− 0.15 to 0.79		
*Vascular invasion*														
Yes	0	–	0.100	0	–	0.033	0.025	0	–	0.734	0	–	0.809	0.050
No	− 0.05	− 0.16 to 0.06		− 0.06	− 0.17 to 0.06			− 0.11	− 0.39 to 0.17		0.04	− 0.24 to 0.32		
Not reported	0.18	− 0.06 to 0.42		0.22	− 0.00 to 0.45			− 0.08	− 0.94 to 0.79		0.24	− 0.53 to 1.01		

*Test for trend

^†^∆Ki67_2week_ = ln((Ki67_2week_ + 0.1)/(ln(Ki67_baseline_ + 0.1))

Adjusted critical values calculated using Benjamini Hochberg method. Significant *p* values following adjustment are highlighted in bold

We also explored in what is a non-randomised comparison whether each of the AIs received was differentially associated with ∆Ki67_2week_. Of patients with paired Ki67_baseline_ and Ki67_2week_; 839 (33%) patients were known to have received anastrozole and 1689 (67%) letrozole. Although considerable change in Ki67 was seen for each AI the median suppression was observed to be slightly less with anastrozole than letrozole (75.6% vs 80.6%, *p* < 0.001, respectively Additional file 3: Fig. S3b) in HER2-ve patients but not in HER2 + ve patients where median suppression did not differ by type of AI (56.5% vs 50.6% respectively, *p* = 0.791). Upon further exploration, the association remained after adjustment for sample type but the difference appeared to be evident only within excision samples but not core-cuts (Additional file 3: Fig. S3b). Inclusion of AI and sample type in multivariable models did not impact the association with other baseline characteristics (Additional file 4: Table S3).

### Complete cell cycle arrest (CCCA), AI group

Suppression of Ki67 to ≤ 2.7% has been used to define CCCA. Additional file  4: Table S4 shows the frequency of CCCA according to the choice of AI and surgical sample type by HER2 status. Similar to analyses of ∆Ki67_2week_, in HER2-ve patients there was a greater likelihood of recording CCCA if the surgical sample was an excision rather than a core-cut (55.4% vs. 44.2%, respectively; *p* < 0.001). There was no difference in the frequency of CCCA according to AI used for core-cuts at 2 weeks (anastrozole 44.8%, letrozole 44.1%). In patients with an excision at 2 weeks, CCCA was significantly less frequent with anastrozole than with letrozole (49.7% vs. 59.1%, respectively; *p* < 0.001). No differences were observed by AI or sample type in the HER2 + ve population but sample size in this subcohort is restrictive.

## Discussion

Ki67 is the most widely measured marker of proliferation in primary breast cancer. While there have been many reports of the association of Ki67 with clinico-pathological parameters in breast cancer there have been very few large studies that focussed entirely on ER + disease where its measurement has greatest impact. The magnitude of our study enabled us not only to confirm previously hypothesised relationships but also to quantify the degree of independence of each relationship within a multivariable context. It also allowed us to discover with high levels of confidence other relationships that have remained either unknown or less clear in earlier studies. We were able to do so for 3 measurements with distinct clinical relationships with clinical outcome: (i) Ki67_baseline_ which is related to prognosis in the absence of treatment[1]; (ii) Ki67_2week_ which relates to the prognosis of patients on adjuvant endocrine therapy otherwise known as residual risk[10, 12]; (iii) ∆Ki67_2week_ which reflects the antiproliferative impact of oestrogen deprivation with an AI and has been shown to predict the relative benefit of endocrine therapies given as adjuvant treatment[3, 7]. While Ki67_baseline_ is often measured in clinical practise for its prognostic information it is not currently considered to have sufficient clinical utility for that purpose to be mandated by authoritative guidelines. However, FDA has recently approved the use of the CDK4/6 inhibitor abemaciclib for use in early breast cancer patients with one of the conditions being that Ki67_baseline_ is > 20%. This enhances the relevance of the data we present here from our large cohort of baseline samples.

Other strengths of the study include the central analysis of Ki67 using a scoring method that was marginally modified prior to its analytical validation by the International Ki67 in Breast Cancer Working Group[13]. Several scorers were involved with a rigorous internal QC programme. The involvement of a large number of hospital sites with variability in collection and fixation procedures might be considered a weakness. On the other hand, the authors view the large number of sites as a strength in that it enables interpretation within the context of routine conduct of Ki67 measurements and allowed the characterisation of an important difference in scores between biopsy types. The study involved only postmenopausal patients and may not be representative of premenopausal cases.

Relationships of Ki67_baseline_ in an ER + population with PgR and HER2 status are well known. We were also able to confirm results from our earlier much smaller patient series[16] that HER2 impedes the antiproliferative response (from approximately 80% to 50%) to AI but does not preclude it. Ellis et al. similarly reported that Ki67 suppression by AIs was less in HER2 + cases[17]. The size of the POETIC trial allows analyses to identify the molecular features that are associated with antiproliferative response or not within the HER2 + population that makes up only about 10% of ER + breast cancer[18].

There was less proportional suppression of Ki67 in PgR- than PgR + cases leading to the relative difference in Ki67_baseline_ between these subsets also being seen at 2 weeks. This is consistent with our earlier report[14] and that of others and suggests that AIs may have greater relative benefit in PgR + than PgR- patients. This has not been detected directly in adjuvant trials but the data from those trials relates to the comparative benefit from AIs versus tamoxifen[19]. The lower value of Ki67_2week_ in the PgR + group is consistent with the substantially better prognosis of such patients on endocrine therapy[20–22]. In contrast, lobular cancers showed a similar suppression of Ki67 compared to ductal cancers suggesting a similar biological response to AIs but better prognosis because of their lower Ki67_baseline_ and Ki67_2week_.

The poorer ∆Ki67_2week_ in higher grade tumours or those with high Ki67, similarly to that in PgR- and HER2 + tumours indicates that those with biologically more aggressive disease but not higher stage disease (cf the data on tumour size and nodal status) have a poorer biologic response to oestrogen deprivation. In our report[23] of whole exome sequencing in samples from POETIC those cases with high mutational load and/or TP53 mutation also had lower ∆Ki67_2week_ and similarly would be enriched for cases with more aggressive disease.

While others have reported lower Ki67 values in excisions versus core-cuts of breast cancers[24, 25] this has not been universally reported[26]. The lack of difference between Ki67 measured at baseline and 2-week in controls where core-cut biopsies were available supports there being little overall impact of the procedures in the trial up to the point of taking the 2-week sample. There may be a number of explanations for the finding that there was a significant difference between Ki67 measured at baseline and then at 2 weeks in controls where the 2-week sample was taken from the surgical resection specimen. Nuclear integrity may be poorly preserved in routinely fixed excision specimens due to a delay in formalin reaching the centre of the excision specimens where the tumour is situated, usually surrounded by a margin of normal tissue which is variable from specimen to specimen. This problem does not occur in core-cuts because of their smaller size. Also, under ultrasound biopsy the needle is placed right at the edge of the tumour or even in it and therefore there is much more rapid fixation of the tumour. Further explanation may be that core biopsies are placed in fixative much more swiftly, indeed almost immediately and the tissue is therefore not exposed to any ischaemic warm time. In contrast wide local excision specimens, mastectomy and mastectomy and en-bloc axillary clearances have on average a greater warm ischaemic time due to the increasing duration of surgical time and ischaemia of the tissues resected. It is also possible that core-cuts may tend to sample more proliferative areas of the tumour although that seems unlikely given that higher staining areas of Ki67 are more commonly found at the tumour edge. Our scoring method involved selection of areas for scoring to represent any heterogeneity in staining but it cannot be completely ruled out that this may also have contributed to the lower values in excisions. Whatever the cause(s) the relative difference of approximately 20% is important to consider and is highly preferable to avoid in presurgical studies. In the absence of a control arm, a presurgical study in which excision specimens are used as the on-treatment sample may artifactually enhance the apparent antiproliferative impact of a treatment. For example, in our study, in the POAI group the median percentage change of Ki67 was -72.6% when the surgical sample was a core-cut compared to -79.3% in excisions. However, as a difference had been observed in the control arm, Ki67_2week_ scores were adjusted for sample type prior to primary analysis by increasing Ki67_2week_ scores derived from a resection sample by 15%. In addition such differences will be essential to consider in the application of cut-offs for Ki67. It is possible that some staining procedures may be more sensitive to differences to variability in fixation; it may therefore be prudent for pathologists to assess the impact of fixation quality on Ki67 analysis within their own practise. We have previously reported the impact of short-term AI therapy on grade and this should not be ignored[12]. Where an AI has been given in the presurgical or neoadjuvant setting preference may well be given to assessment of grade from a core rather than excision specimen to minimise this impact.

The suppression of Ki67 by AIs was similar to that reported previously[3, 7] but the suggestion of an apparent statistically significant difference between letrozole and anastrozole in the degree of suppression has not been previously reported. Although type of AI remains significant when adjusting for other clinico-pathological factors, it is important to note that this is not a randomised comparison but the choice of AI was centre dependent influenced by local clinical practice. Given the difference is only observed in excision samples and not core-cuts and only in HER2-ve tumours, there is a high probability that this difference may be related to unmeasured or artefactual differences—e.g. in surgical procedures or processing of surgical specimens between centres. There was no difference in clinical outcomes between these two AIs in randomised clinical trials either in advanced breast cancer or in primary ER + breast cancer[27, 28]. There is therefore no evidence for a difference in clinical efficacy of these two agents in spite of a known small difference in estradiol suppression and the Ki67 data reported in this manuscript.

## Conclusions

In conclusion, the magnitude of this study allowed assessment of relationships between clinico-pathological factors and Ki67_baseline_, POAI-induced and untreated ∆Ki67_2week_ and Ki67_2week_ with high degrees of confidence, in particular, illustrating that POAI-induced ∆Ki67_2week_ was independent of tumour size, nodal involvement, histology and vascular invasion but associated with both grade and PgR status. Lower values of Ki67 occur when measured on excision specimens rather than core-cut biopsies, and both these factors should be considered when either ∆Ki67_2week_ or Ki67_2week_ are used in routine clinical practice to aid treatment decisions or in clinical trials to assess new drug therapies. Our recommendation would be to use core-core comparisons where possible with the second core being taken in situ as soon as the tumour is excised to avoid this artefact.

## Supplementary Information

**Additional file 1**: **Fig. S1**. Distribution of Ki67_Baseline_ a. Distribution of Ki67 % positive cells b. Distribution of ln(Ki67+0.1) c. Distribution of Ki67_Baseline_ by HER2 status

**Additional file 2**: **Fig. S2**. Distribution of A. log(Ki67_Baseline_ +0.1) for all patients, B. log(Ki672_week_ +0.1) in patients allocated control, C. log fold. change Ki67 in patients allocated control, D. log(Ki67_2week_ +0.1) in patients allocated AI and E. log fold change Ki67 in patients allocated AI. Presented separately for HER2- and HER2+ patients.

**Additional file 3**: **Fig. S3**. Log fold change in Ki67 A. for patients allocated control by sample. type, B. for patients allocated AI by sample type and choice of AI. Presented separately for HER2- and HER2- patients.

**Additional file 4**: **Table S1**. Univariable and multivariable linear regression results for change in Ki67 in patients allocated to control by HER2 status. **Table S2**. Multivariable linear regression results for change in Ki67 in patients allocated to control by HER2 status showing adjustment for sample type. **Table S3**. Multivariable linear regression results for change in Ki67 in patients allocated to AI by HER2 status showing adjustment for sample type and AI choice. **Table S4**. CCCA by AI and sample type in patients allocated AI.

## Data Availability

De-identified data will be made available to other researchers on request, subject to approval of a formal data access request in accordance with the ICR-CTSU data and sample access policy. Trial documentation including the protocol are available on request by contacting poetic-icrctsu@icr.ac.uk. The ICR-CTSU supports the wider dissemination of information from the research it does, and increased cooperation between investigators. Trial data is collected, managed, stored, shared, and archived according to ICR-CTSU Standard Operating Procedures in order to ensure the enduring quality, integrity, and utility of the data. Formal requests for data sharing are considered in line with the Institute of Cancer Research Clinical Trials and Statistics Unit (ICR-CTSU) procedures with due regard given to funder and sponsor guidelines. Requests are via a standard proforma describing the nature of the proposed research and extent of data requirements. Data recipients are required to enter a formal data sharing agreement which describes the conditions for release and requirements for data transfer, storage, archiving, publication and intellectual property. Requests are reviewed by the Trial Management Group (TMG) in terms of scientific merit and ethical considerations including patient consent. Data sharing is allowed if proposed projects have a sound scientific or patient benefit rationale as agreed by the TMG and approved by the Trial Steering Committee as required. Restrictions relating to patient confidentiality and consent will be limited by aggregating and anonymising identifiable patient data. Additionally all indirect identifiers that might lead to deductive disclosures will be removed in line with Cancer Research UK Data Sharing Guidelines. Additional documents might be shared if approved by the TMG and Trial Steering Committee (eg., statistical analysis plan and informed consent form).
