# Supplementary material for: Clinico-pathologic relationships with Ki67 and its change with short-term aromatase inhibitor treatment in primary ER + breast cancer: further results from the POETIC trial (CRUK/07/015)
Source: Breast Cancer Res. 2023 Apr 12;25:39. doi: 10.1186/s13058-023-01626-3 (PMC10099675; doi:10.1186/s13058-023-01626-3)
Supplement: Supplementary file 1 — Additional file 1: Fig. S1. Distribution of Ki67Baseline a. Distribution of Ki67 % positive cells b. Distribution of ln(Ki67+0.1) c. Distribution of Ki67Baseline by HER2 status [file 13058_2023_1626_MOESM1_ESM.pdf]

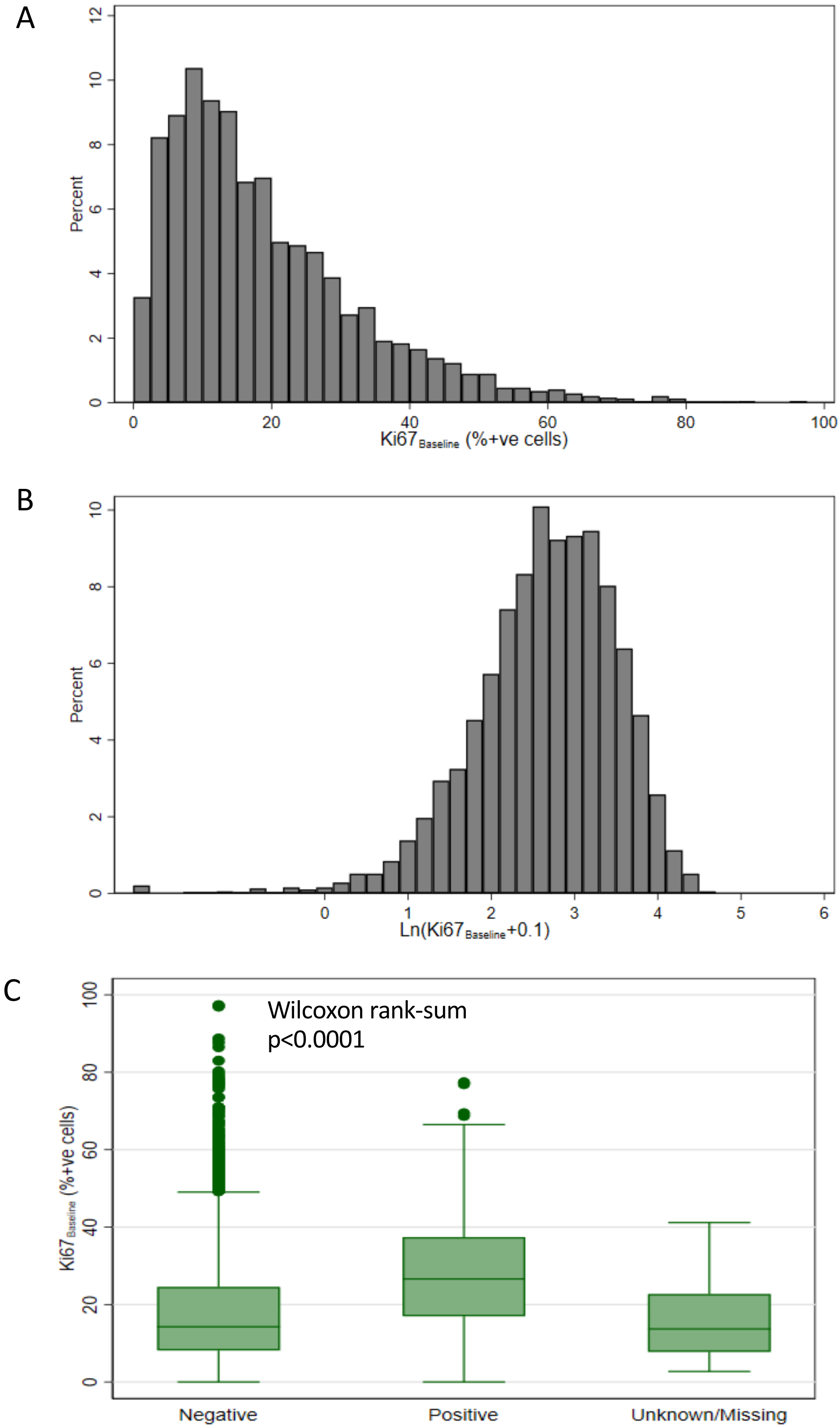

Supplementary figure 1. Distribution of $Ki67_{Baseline}$

a. Distribution of Ki67 % positive cells

b. Distribution of ln(ki67+0.1)

c. Distribution of $Ki67_{Baseline}$ by HER2 status
