# Supplementary material for: Clinico-pathologic relationships with Ki67 and its change with short-term aromatase inhibitor treatment in primary ER + breast cancer: further results from the POETIC trial (CRUK/07/015)
Source: Breast Cancer Res. 2023 Apr 12;25:39. doi: 10.1186/s13058-023-01626-3 (PMC10099675; doi:10.1186/s13058-023-01626-3)
Supplement: Supplementary file 2 — Additional file 2: Fig. S2. Distribution of A. log(Ki67Baseline +0.1) for all patients, B. log(Ki672week +0.1) in patients allocated control, C. log fold. change Ki67 in patients allocated control, D. log(Ki672week +0.1) in patients allocated AI and E. log fold change Ki67 in patients allocated AI. Presented separately for HER2- and HER2+ patients. [file 13058_2023_1626_MOESM2_ESM.pdf]

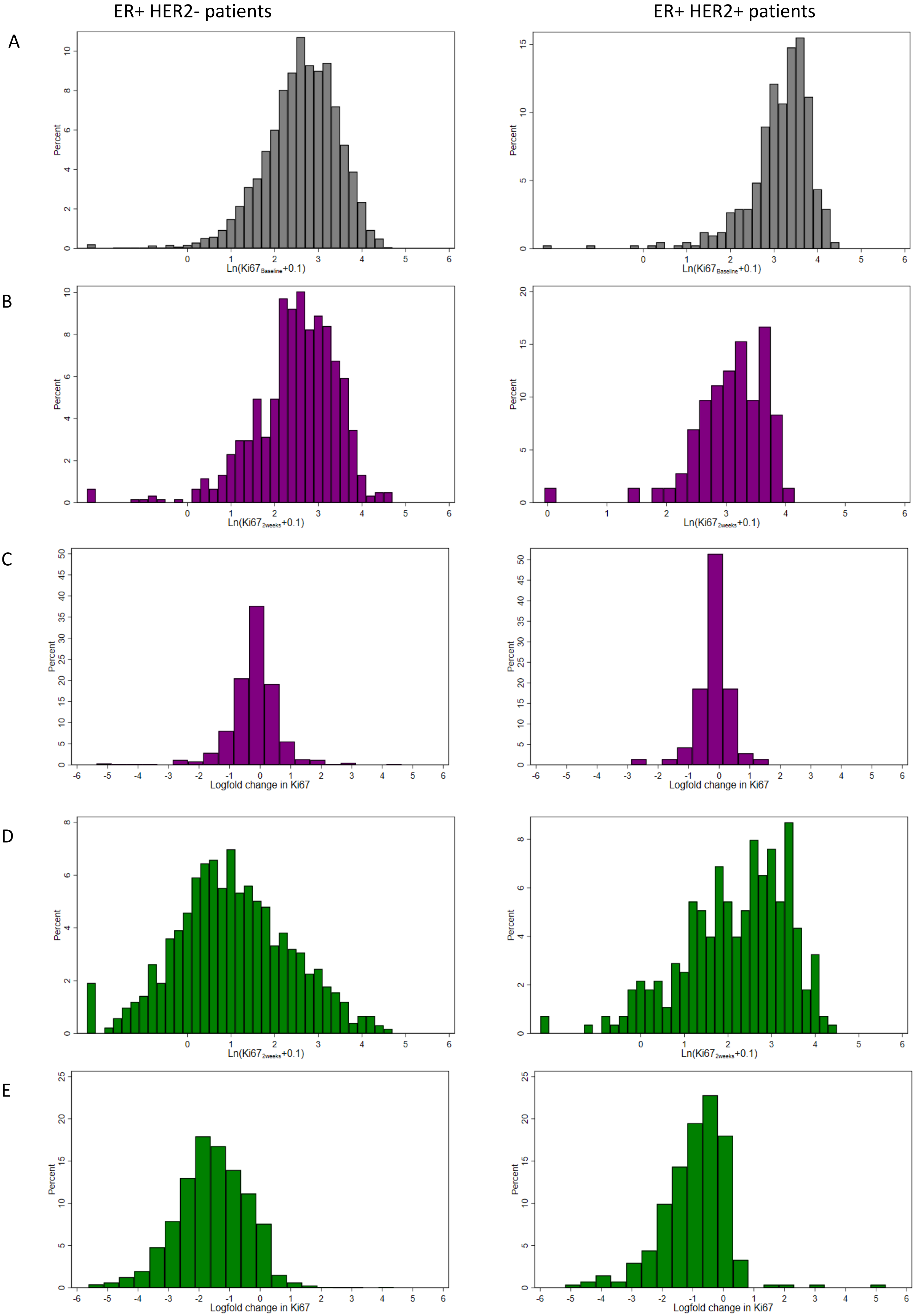

Supplementary figure 2.
Distribution of A. log($Ki67_{Baseline}$ +0.1) for all patients, B. log($Ki67_{2week}$ +0.1) in patients allocated control, C. log fold change Ki67 in patients allocated control, D. log($Ki67_{2week}$ +0.1) in patients allocated AI and E. log fold change Ki67 in patients allocated AI. Presented separately for HER2- and HER2+ patients.
