# Supplementary material for: Clinico-pathologic relationships with Ki67 and its change with short-term aromatase inhibitor treatment in primary ER + breast cancer: further results from the POETIC trial (CRUK/07/015)
Source: Breast Cancer Res. 2023 Apr 12;25:39. doi: 10.1186/s13058-023-01626-3 (PMC10099675; doi:10.1186/s13058-023-01626-3)
Supplement: Supplementary file 3 — Additional file 3: Fig. S3. Log fold change in Ki67 A. for patients allocated control by sample. type, B. for patients allocated AI by sample type and choice of AI. Presented separately for HER2- and HER2- patients. [file 13058_2023_1626_MOESM3_ESM.pdf]

A

B

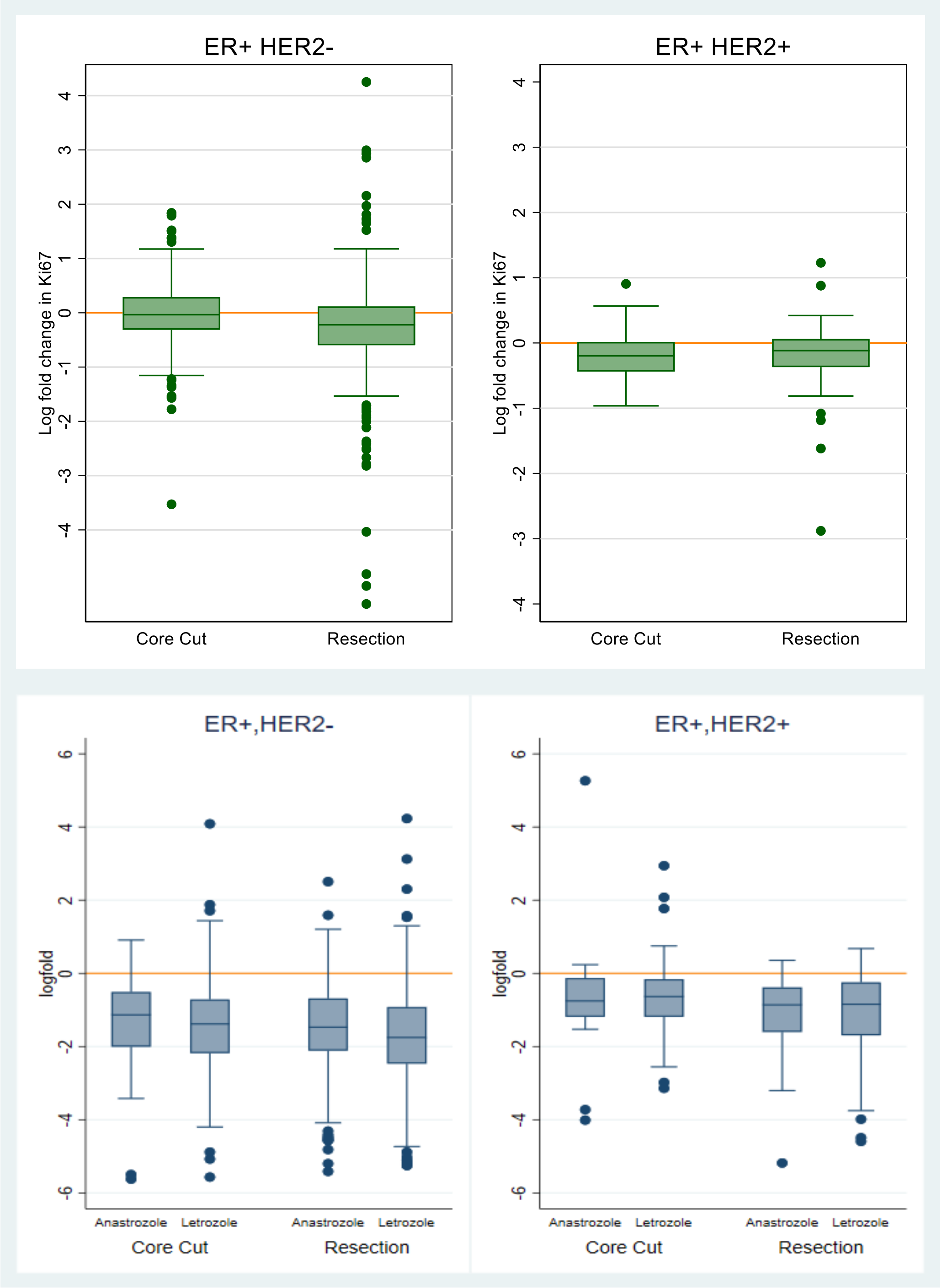

Supplementary figure 3.
Log fold change in Ki67 A. for patients allocated control by sample type, B. for patients allocated AI by sample type and choice of AI. Presented separately for HER2- and HER2- patients.
