# Supplementary material for: Clinico-pathologic relationships with Ki67 and its change with short-term aromatase inhibitor treatment in primary ER + breast cancer: further results from the POETIC trial (CRUK/07/015)
Source: Breast Cancer Res. 2023 Apr 12;25:39. doi: 10.1186/s13058-023-01626-3 (PMC10099675; doi:10.1186/s13058-023-01626-3)
Supplement: Supplementary file 4 — Additional file 4: Table S1. Univariable and multivariable linear regression results for change in Ki67 in patients allocated to control by HER2 status. Table S2. Multivariable linear regression results for change in Ki67 in patients allocated to control by HER2 status showing adjustment for sample type. Table S3. Multivariable linear regression results for change in Ki67 in patients allocated to AI by HER2 status showing adjustment for sample type and AI choice. Table S4. CCCA by AI and sample type in patients allocated AI. [file 13058_2023_1626_MOESM4_ESM.docx]

Supplementary table 1. Univariable and multivariable linear regression results for change in Ki67 in patients allocated to control by HER2 status

|  | | **ER+ HER2-** | | | | | | | **ER+ HER2+** | | | | | | |
| --- | --- | --- | --- | --- | --- | --- | --- | --- | --- | --- | --- | --- | --- | --- | --- |
|  | | **Univariable** | | | **Multivariable** | | | | **Univariable** | | | **Multivariable** | | | |
|  |  | **β** | **ci** | **p-value** | **β** | **ci** | **p-value** | **Adjusted critical value** | **β** | **ci** | **p-value** | **β** | **ci** | **p-value** | **Adjusted critical value** |
| Baseline Ki67 (log) | | -0.34 | -0.42 - -0.27 | <0.001 | -0.43 | -0.51 - -0.35 | **<0.001** | **0.006** | -0.29 | -0.55 - -0.03 | 0.027 | -0.27 | -0.57 - 0.02 | 0.036 | 0.006 |
| PgR Status | Positive | 0 | - | 0.713 | 0 | - | 0.126 | 0.031 | 0 | - | 0.958 | 0 | - | 0.843 | 0.050 |
|  | Negative | 0.03 | -0.20 - 0.26 |  | 0.09 | -0.12 - 0.30 |  |  | 0.05 | -0.31 - 0.40 |  | 0.08 | -0.34 - 0.49 |  |  |
|  | Unknown | -0.06 | -0.21 - 0.10 |  | -0.12 | -0.27 - 0.03 |  |  | -0.00 | -0.33 - 0.33 |  | -0.04 | -0.42 - 0.34 |  |  |
| Tumour grade (baseline) | 1 | 0 | - | 0.719 |  |  |  |  | 0 | - | 0.328 |  |  |  |  |
|  | 2 | 0.05 | -0.14 - 0.25 |  |  |  |  |  | 0.01 | -1.14 - 1.15 |  |  |  |  |  |
|  | 3 | -0.01 | -0.25 - 0.23 |  |  |  |  |  | -0.04 | -1.19 - 1.11 |  |  |  |  |  |
|  | Not known | -0.07 | -0.34 - 0.21 |  |  |  |  |  | 0.51 | -0.76 - 1.77 |  |  |  |  |  |
| Tumour grade (2week) | 1 | 0 | - | 0.718  (0.836)* | 0 | - | **<0.001** | **0.013** | 0 | - | 0.798  (0.172)* | 0 | - | 0.135 | 0.013 |
|  | 2 | 0.05 | -0.17 - 0.27 |  | 0.23 | 0.03 - 0.44 |  |  | 0.24 | -0.93 - 1.41 |  | 0.39 | -0.92 - 1.71 |  |  |
|  | 3 | 0.09 | -0.14 - 0.33 |  | 0.56 | 0.32 - 0.80 |  |  | 0.31 | -0.85 - 1.47 |  | 0.65 | -0.65 - 1.96 |  |  |
| Tumour size (baseline) | 0-2cm | 0 | - | 0.358  (0.567)* |  |  |  |  | 0 | - | 0.053  (0.640)* |  |  |  |  |
|  | 2-5cm | -0.10 | -0.24 - 0.04 |  |  |  |  |  | -0.18 | -0.45 - 0.08 |  |  |  |  |  |
|  | >5cm | -0.08 | -0.61 - 0.45 |  |  |  |  |  | 1.02 | -0.10 - 2.13 |  |  |  |  |  |
| Tumour size (2week) | 0-2cm | 0 | - | 0.077  (0.003)* | 0 | - | 0.425 | 0.044 | 0 | - | 0.783  (0.417)* | 0 | - | 0.570 | 0.038 |
|  | 2-5cm | -0.13 | -0.26 - 0.01 |  | -0.08 | -0.21 - 0.06 |  |  | -0.03 | -0.32 - 0.26 |  | -0.15 | -0.48 - 0.19 |  |  |
|  | >5cm | -0.29 | -0.60 - 0.03 |  | -0.16 | -0.47 - 0.16 |  |  | 0.15 | -0.36 - 0.66 |  | 0.02 | -0.64 - 0.67 |  |  |
| Histological type (baseline) | Ductal | 0 | - | 0.085 |  |  |  |  | 0 | - | 0.635 |  |  |  |  |
|  | Lobular | -0.05 | -0.25 - 0.15 |  |  |  |  |  | -0.00 | -0.43 - 0.43 |  |  |  |  |  |
|  | Other | 0.40 | 0.03 - 0.76 |  |  |  |  |  | 0.38 | -0.44 - 1.20 |  |  |  |  |  |
| Histological type (2week) | Ductal | 0 | - | 0.065 | 0 | - | 0.093 | 0.019 | 0 | - | 0.546 | 0 | - | 0.548 | 0.025 |
|  | Lobular | -0.18 | -0.37 - 0.01 |  | -0.18 | -0.36 - 0.00 |  |  | -0.14 | -0.59 - 0.32 |  | -0.15 | -0.72 - 0.42 |  |  |
|  | Other | 0.22 | -0.13 - 0.57 |  | 0.13 | -0.21 - 0.47 |  |  | NA | - |  | NA | - |  |  |
| Nodal status | N0 | 0 | - | 0.030  (0.253)* | 0 | - | 0.100 | 0.025 | 0 | - | 0.335  (0.441)* | 0 | - | 0.576 | 0.044 |
|  | N1-3 | 0.04 | -0.11 - 0.19 |  | 0.10 | -0.05 - 0.25 |  |  | 0.21 | -0.10 - 0.52 |  | 0.13 | -0.23 - 0.49 |  |  |
|  | N4+ | -0.28 | -0.51 - -0.06 |  | -0.14 | -0.38 - 0.09 |  |  | 0.16 | -0.20 - 0.52 |  | 0.18 | -0.28 - 0.64 |  |  |
| Age group | <60 | 0.09 | -0.10 - 0.27 | 0.670  (0.657)* | 0.05 | -0.13 - 0.22 | 0.399 | 0.038 | 0.35 | -0.01 - 0.72 | 0.214  (0.343)* | 0.36 | -0.09 - 0.81 | 0.200 | 0.019 |
|  | 60-69 | 0 | - |  | 0 | - |  |  | 0 | - |  | 0 | - |  |  |
|  | 70-79 | 0.06 | -0.11 - 0.22 |  | 0.10 | -0.05 - 0.25 |  |  | 0.05 | -0.28 - 0.39 |  | 0.08 | -0.31 - 0.47 |  |  |
|  | 80+ | -0.04 | -0.27 - 0.19 |  | -0.07 | -0.29 - 0.14 |  |  | -0.10 | -0.60 - 0.39 |  | -0.21 | -0.76 - 0.33 |  |  |
| Vascular invasion | Yes | 0 | - | 0.647 | 0 | - | 0.921 | 0.050 | 0 | - | 0.810 | 0 | - | 0.555 | 0.031 |
|  | No | 0.07 | -0.08 - 0.22 |  | 0.01 | -0.15 - 0.16 |  |  | -0.03 | -0.31 - 0.25 |  | 0.04 | -0.28 - 0.37 |  |  |
|  | Not reported | -0.01 | -0.39 - 0.37 |  | -0.06 | -0.43 - 0.30 |  |  | -0.26 | -1.10 - 0.57 |  | -0.39 | -1.31 - 0.53 |  |  |

**Test for trend*

*Adjusted critical values calculated using Benjamini Hochberg method. Significant p-values following adjustment are highlighted in bold*

Supplementary table 2. Multivariable linear regression results for change in Ki67 in patients allocated to control by HER2 status showing adjustment for sample type

|  | | **ER+ HER2-** | | | | | | | **ER+ HER2+** | | | | | | |
| --- | --- | --- | --- | --- | --- | --- | --- | --- | --- | --- | --- | --- | --- | --- | --- |
|  | | **Without Sample Type** | | | **Including Sample Type** | | | | **Without Sample Type** | | | **Including Sample Type** | | | |
|  |  | **β** | **ci** | **p-value** | **β** | **ci** | **p-value** | **Adjusted critical value** | **β** | **ci** | **p-value** | **β** | **ci** | **p-value** | **Adjusted critical value** |
| Sample type | Core cut |  |  |  | 0 | - | **<0.001** | **0.017** |  |  |  | 0 | - | 0.550 | 0.028 |
|  | Resection |  |  |  | -0.27 | -0.41 - -0.14 |  |  |  |  |  | -0.10 | -0.49 – 0.29 |  |  |
| Baseline Ki67 (log) | | -0.43 | -0.51 - -0.35 | **<0.001** | -0.44 | -0.52 - -0.35 | **<0.001** | **0.006** | -0.27 | -0.57 - 0.02 | 0.036 | -0.27 | -0.56 – 0.03 | 0.040 | 0.006 |
| PgR Status | Positive | 0 | - | 0.126 | 0 | - | 0.254 | 0.033 | 0 | - | 0.843 | 0 | - | 0.810 | 0.050 |
|  | Negative | 0.09 | -0.12 - 0.30 |  | 0.08 | -0.13 – 0.29 |  |  | 0.08 | -0.34 - 0.49 |  | 0.07 | -0.34 – 0.49 |  |  |
|  | Unknown | -0.12 | -0.27 - 0.03 |  | -0.09 | -0.24 – 0.05 |  |  | -0.04 | -0.42 - 0.34 |  | -0.06 | -0.46 – 0.34 |  |  |
| Tumour grade (2week) | 1 | 0 | - | **<0.001** | 0 | - | **<0.001** | **0.011** | 0 | - | 0.135 | 0 | - | 0.143 | 0.011 |
|  | 2 | 0.23 | 0.03 - 0.44 |  | 0.22 | 0.01 – 0.42 |  |  | 0.39 | -0.92 - 1.71 |  | 0.49 | -0.86 – 1.83 |  |  |
|  | 3 | 0.56 | 0.32 - 0.80 |  | 0.54 | 0.30 – 0.78 |  |  | 0.65 | -0.65 - 1.96 |  | 0.73 | -0.60 – 2.06 |  |  |
| Tumour size (2week) | 0-2cm | 0 | - | 0.425 | 0 | - | 0.395 | 0.039 | 0 | - | 0.570 | 0 | - | 0.600 | 0.039 |
|  | 2-5cm | -0.08 | -0.21 - 0.06 |  | -0.08 | -0.21 – 0.06 |  |  | -0.15 | -0.48 - 0.19 |  | -0.15 | -0.49 – 0.20 |  |  |
|  | >5cm | -0.16 | -0.47 - 0.16 |  | -0.17 | -0.48 – 0.14 |  |  | 0.02 | -0.64 - 0.67 |  | -0.02 | -0.68 – 0.64 |  |  |
| Histological type (2week) | Ductal | 0 | - | 0.093 | 0 | - | 0.088 | 0.022 | 0 | - | 0.548 | 0 | - | 0.614 | 0.044 |
|  | Lobular | -0.18 | -0.36 - 0.00 |  | -0.18 | -0.36 - -0.00 |  |  | -0.15 | -0.72 - 0.42 |  | -0.13 | -0.70 – 0.45 |  |  |
|  | Other | 0.13 | -0.21 - 0.47 |  | 0.12 | -0.21 – 0.46 |  |  | NA | - |  | NA | - |  |  |
| Nodal status | N0 | 0 | - | 0.100 | 0 | - | 0.111 | 0.028 | 0 | - | 0.576 | 0 | - | 0.477 | 0.022 |
|  | N1-3 | 0.10 | -0.05 - 0.25 |  | 0.09 | -0.06 – 0.25 |  |  | 0.13 | -0.23 - 0.49 |  | 0.17 | -0.21 – 0.54 |  |  |
|  | N4+ | -0.14 | -0.38 - 0.09 |  | -0.14 | -0.38 – 0.09 |  |  | 0.18 | -0.28 - 0.64 |  | 0.18 | -0.28 – 0.65 |  |  |
| Age group | <60 | 0.05 | -0.13 - 0.22 | 0.399 | 0.03 | -0.14 – 0.20 | 0.410 | 0.044 | 0.36 | -0.09 - 0.81 | 0.200 | 0.37 | -0.08 – 0.83 | 0.164 | 0.017 |
|  | 60-69 | 0 | - |  | 0 | - |  |  | 0 | - |  | 0 | - |  |  |
|  | 70-79 | 0.10 | -0.05 - 0.25 |  | 0.06 | -0.09 – 0.21 |  |  | 0.08 | -0.31 - 0.47 |  | 0.11 | -0.29 – 0.50 |  |  |
|  | 80+ | -0.07 | -0.29 - 0.14 |  | -0.13 | -0.34 – 0.09 |  |  | -0.21 | -0.76 - 0.33 |  | -0.22 | -0.78 – 0.33 |  |  |
| Vascular invasion | Yes | 0 | - | 0.921 | 0 | - | 0.966 | 0.050 | 0 | - | 0.555 | 0 | - | 0.561 | 0.033 |
|  | No | 0.01 | -0.15 - 0.16 |  | -0.00 | -0.16 – 0.15 |  |  | 0.04 | -0.28 - 0.37 |  | 0.01 | -0.32 – 0.34 |  |  |
|  | Not reported | -0.06 | -0.43 - 0.30 |  | -0.05 | -0.41 – 0.32 |  |  | -0.39 | -1.31 - 0.53 |  | -0.42 | -1.34 – 0.51 |  |  |

Supplementary table 3. Multivariable linear regression results for change in Ki67 in patients allocated to AI by HER2 status showing adjustment for sample type and AI choice

|  | | **ER+ HER2-** | | | | | | | **ER+ HER2+** | | | | | | |
| --- | --- | --- | --- | --- | --- | --- | --- | --- | --- | --- | --- | --- | --- | --- | --- |
|  | | **Without Sample Type or AI** | | | **Including Sample Type and AI** | | | | **Without Sample Type or AI** | | | **Including Sample Type and AI** | | | |
|  |  | **β** | **ci** | **p-value** | **β** | **ci** | **p-value** | **Adjusted critical value** | **β** | **ci** | **p-value** | **β** | **ci** | **p-value** | **Adjusted critical value** |
| Sample Type | Core-cut |  |  |  | 0 | - | **<0.001** | **0.020** |  |  |  | 0 | - | 0.039 | 0.020 |
|  | Resection |  |  |  | -0.27 | -0.37 - -0.16 |  |  |  |  |  | -0.28 | -0.56 - -0.00 |  |  |
| AI Type | Anastrozole |  |  |  | 0 | - | **<0.001** | **0.025** |  |  |  | 0 | - | 0.190 | 0.035 |
|  | Letrozole |  |  |  | -0.22 | -0.32 - -0.12 |  |  |  |  |  | -0.17 | -0.44 – 0.10 |  |  |
| Baselin Ki67 (log) | | -0.41 | -0.47 - -0.35 | **<0.001** | -0.42 | -0.47 - -0.36 | **<0.001** | **0.005** | -0.61 | -0.77 - -0.46 | **<0.001** | -0.60 | -0.75 - -0.44 | **<0.001** | **0.005** |
| PgR Status | Positive | 0 | - | **<0.001** | 0 | - | **<0.001** | **0.015** | 0 | - | **0.008** | 0 | - | 0.017 | 0.015 |
|  | Negative | 0.45 | 0.30 - 0.60 |  | 0.45 | 0.30 – 0.60 |  |  | 0.45 | 0.15 - 0.75 |  | 0.41 | 0.11 – 0.71 |  |  |
|  | Unknown | 0.11 | 0.01 - 0.22 |  | 1.15 | -0.03 – 0.18 |  |  | 0.07 | -0.22 - 0.36 |  | 0.06 | -0.25 – 0.36 |  |  |
| Tumour grade (2week) | 1 | 0 | - | **<0.001** | 0 | - | **<0.001** | **0.010** | 0 | - | **<0.001** | 0 | - | **<0.001** | **0.010** |
|  | 2 | 0.23 | 0.10 - 0.37 |  | 0.24 | 0.10 – 0.37 |  |  | 0.32 | -0.34 - 0.97 |  | 0.35 | -0.30 – 1.00 |  |  |
|  | 3 | 1.16 | 0.97 - 1.34 |  | 1.15 | 0.96 – 1.33 |  |  | 1.14 | 0.45 - 1.82 |  | 1.20 | 0.51 – 1.88 |  |  |
| Tumour size (2week) | 0-2cm | 0 | - | 0.525 | 0 | - | 0.563 | 0.045 | 0 | - | 0.201 | 0 | - | 0.185 | 0.030 |
|  | 2-5cm | -0.03 | -0.13 - 0.07 |  | -0.03 | -0.13 – 0.07 |  |  | 0.23 | -0.03 - 0.50 |  | 0.24 | -0.03 – 0.50 |  |  |
|  | >5cm | -0.13 | -0.36 - 0.10 |  | -0.12 | -0.35 – 0.11 |  |  | 0.19 | -0.42 - 0.80 |  | 0.21 | -0.39 – 0.82 |  |  |
| Histological type (2week) | Ductal | 0 | - | 0.091 | 0 | - | 0.110 | 0.035 | 0 | - | 0.624 | 0 | - | 0.689 | 0.045 |
|  | Lobular | -0.14 | -0.27 - -0.01 |  | -0.14 | -0.27 - -0.01 |  |  | -0.16 | -0.69 - 0.37 |  | -0.15 | -0.67 – 0.38 |  |  |
|  | Other | 0.06 | -0.17 - 0.29 |  | 0.03 | -0.19 – 0.26 |  |  | -0.37 | -1.36 - 0.62 |  | -0.32 | -1.31 – 0.67 |  |  |
| Nodal status | N0 | 0 | - | 0.880 | 0 | - | 0.840 | 0.050 | 0 | - | 0.252 | 0 | - | 0.371 | 0.040 |
|  | N1-3 | 0.01 | -0.10 - 0.12 |  | 0.01 | -0.09 – 0.12 |  |  | -0.09 | -0.38 - 0.21 |  | -0.05 | -0.35 – 0.24 |  |  |
|  | N4+ | -0.03 | -0.20 - 0.13 |  | -0.04 | -0.20 – 0.13 |  |  | 0.23 | -0.14 - 0.59 |  | 0.21 | -0.16 – 0.58 |  |  |
| Age group | <60 | 0.10 | -0.02 - 0.23 | 0.292 | 0.11 | -0.01 – 0.24 | 0.273 | 0.040 | 0.34 | 0.02 - 0.66 | 0.130 | 0.36 | 0.04 – 0.68 | 0.133 | 0.025 |
|  | 60-69 | 0 | - |  | 0 | - |  |  | 0 | - |  | 0 | - |  |  |
|  | 70-79 | 0.00 | -0.11 - 0.12 |  | 0.01 | -0.11 – 0.12 |  |  | 0.08 | -0.21 - 0.38 |  | 0.11 | -0.18 – 0.40 |  |  |
|  | 80+ | 0.09 | -0.07 - 0.24 |  | 0.07 | -0.09 – 0.22 |  |  | 0.32 | -0.15 - 0.79 |  | 0.24 | -0.23 – 0.72 |  |  |
| Vascular invasion | Yes | 0 | - | 0.033 | 0 | - | 0.068 | 0.030 | 0 | - | 0.809 | 0 | - | 0.863 | 0.050 |
|  | No | -0.06 | -0.17 - 0.06 |  | -0.04 | -0.16 – 0.07 |  |  | 0.04 | -0.24 - 0.32 |  | 0.00 | -0.28 – 0.28 |  |  |
|  | Not reported | 0.22 | -0.00 - 0.45 |  | 0.21 | -0.02 – 0.43 |  |  | 0.24 | -0.53 - 1.01 |  | 0.20 | -0.57 – 0.97 |  |  |

Supplementary table 4. CCCA by AI and sample type in patients allocated AI

|  | **N with Ki67_2week_ value** | **CCCA (Ki67 </=2.7%)** | | **Fisher’s exact p-value** |
| --- | --- | --- | --- | --- |
|  |  | **n** | **%** |  |
| **ER+ HER2-** |  |  |  |  |
| All patients | 2250 | 1179 | 52.4 | - |
|  |  |  |  |  |
| Anastrozole | 750 | 367 | 48.9 | 0.02 |
| Letrozole | 1500 | 812 | 54.1 |  |
|  |  |  |  |  |
| Core-cut | 608 | 269 | 44.2 | <0.001 |
| Resection | 1637 | 907 | 55.4 |  |
|  |  |  |  |  |
| ***Within core-cut:*** |  |  |  |  |
| Anastrozole | 105 | 47 | 44.8 | 0.91 |
| Letrozole | 503 | 222 | 44.1 |  |
|  |  |  |  |  |
| ***Within resection:*** |  |  |  |  |
| Anastrozole | 644 | 320 | 49.7 | <0.001 |
| Letrozole | 993 | 587 | 59.1 |  |
|  |  |  |  |  |
| **ER+ HER2+** |  |  |  |  |
| All patients | 276 | 46 | 16.7 | - |
|  |  |  |  |  |
| Anastrozole | 91 | 14 | 15.4 | 0.73 |
| Letrozole | 185 | 32 | 17.3 |  |
|  |  |  |  |  |
| Core-cut | 83 | 13 | 15.7 | 0.86 |
| Resection | 192 | 33 | 17.2 |  |
|  |  |  |  |  |
| ***Within core-cut:*** |  |  |  |  |
| Anastrozole | 22 | 4 | 18.2 | 0.74 |
| Letrozole | 61 | 9 | 14.8 |  |
|  |  |  |  |  |
| ***Within resection:*** |  |  |  |  |
| Anastrozole | 69 | 10 | 14.5 | 0.55 |
| Letrozole | 123 | 23 | 18.7 |  |
